# Postoperative Empyema Due to *Leclercia adecarboxylata* Following Mesothelioma Surgery: A Case Report

**DOI:** 10.3390/pathogens14020125

**Published:** 2025-01-30

**Authors:** John Fernando Montenegro, Miguel Ángel Diaz-Diaz, Sinthia Vidal-Cañas, Gustavo Urriago, Vanessa Correa, Luis Álvaro Melo-Burbano, Yamil Liscano

**Affiliations:** 1 Grupo de Investigación en Salud Integral (GISI), Departamento Facultad de Salud, Universidad Santiago de Cali, Cali 5183000, Colombia; sinthia.vidal00@usc.edu.co; 2 Programa de Especialización en Medicina Interna, Facultad de Salud, Universidad Santiago de Cali, Cali 5183000, Colombia; miguel1diaz996@gmail.com (M.Á.D.-D.); gustavo.urriago00@usc.edu.co (G.U.); or svcorreaf@gmail.com (V.C.); alvaromelomdint@gmail.com (L.Á.M.-B.); 3 Departamento de Investigación y Educación, Clínica de Occidente S.A., Cali 760046, Colombia

**Keywords:** empyema, *Leclercia adecarboxylata*, immunocompromised host, antibiotic sensitivity tests, case reports

## Abstract

Background/Objectives: *Leclercia adecarboxylata* (*L. adecarboxylata*) is a rare opportunistic pathogen that can cause severe infections like empyema, particularly in immunocompromised individuals. We aim to highlight the importance of the early detection and personalized treatment of *L. adecarboxylata* infections in patients with comorbidities such as malignant mesothelioma. Methods: We present the case of a 57-year-old man with type 2 diabetes mellitus, hypertension, and malignant mesothelioma who developed a parapneumonic effusion that progressed to empyema. After undergoing pleurectomy and pleurodesis, intraoperative cultures identified *L. adecarboxylata*. Targeted antibiotic therapy was initiated based on the culture results, and the patient’s response was closely monitored. Results: The patient responded well to targeted antibiotic therapy with ampicillin/sulbactam following the initial empirical treatment with piperacillin/tazobactam. The identification of *L. adecarboxylata*—a rare finding in empyema cases—was crucial for effective management. The patient recovered fully without complications, highlighting the importance of the early identification and individualized treatment of infections caused by rare pathogens. Conclusions: This case underscores the need to consider *L. adecarboxylata* in immunocompromised patients presenting with unusual infections. Early detection through advanced diagnostic techniques and personalized antibiotic therapy can improve clinical outcomes and help prevent antimicrobial resistance. Increased clinical awareness and further research into the resistance patterns and treatment approaches for *L. adecarboxylata* are essential to enhance patient care.

## 1. Introduction

*Leclercia adecarboxylata* is a bacterium that was initially classified as *Escherichia adecarboxylata* by Leclerc, due to its phenotypic similarity to *Escherichia coli* [1,2]. However, through molecular techniques, it was reclassified as *L. adecarboxylata.* This bacterium plays an opportunistic role in immunocompromised patients, being associated with bacteremia, skin and soft tissue infections, cholecystitis, peritonitis, and pneumonia. In immunocompetent patients, it typically presents as part of polymicrobial infections, whereas in immunocompromised individuals, it can cause monomicrobial infections [1,3,4]. Due to its rarity and underdiagnosis, *L. adecarboxylata* often goes unrecognized, which allows it to acquire multidrug resistance genes, significantly limiting therapeutic options [5]. There have been reports of multidrug-resistant strains producing extended-spectrum β-lactamases (ESBLs), as well as carbapenemases such as NDM-1. Alarmingly, some strains have been found to carry plasmids encoding colistin resistance [6].

Despite the increasing awareness of this pathogen, there is still a lack of comprehensive studies on its global incidence. Most of the existing literature consists of case reports, often associated with healthcare settings, particularly in patients using urinary catheters, ventilators, or receiving total parenteral nutrition [7]. Although *L. adecarboxylata* remains a rare bacterium, it is reportedly susceptible to many antimicrobials, which makes early identification crucial for appropriate management. In Colombia, a case of *L. adecarboxylata* infection was reported in a patient with poorly controlled type 2 diabetes mellitus who developed a soft tissue infection after trauma with plant material [2]. Another case involved a pediatric patient who developed peritonitis due to this organism [8].

What sets this case apart is the association of *L. adecarboxylata* with postoperative empyema, a rare clinical entity. Previously, Shaikhain et al., 2021 [9], reported a case of *L. adecarboxylata* bacteremia in a non-immunosuppressed 74-year-old woman who presented with pneumonia and other comorbidities. The patient initially received treatment with antibiotics but developed bacteremia caused by *L. adecarboxylata* identified through blood cultures. Despite initial complications, the infection responded well to meropenem. However, the patient subsequently developed *Clostridium difficile* infection and died from severe colitis. This case highlighted the importance of considering *L. adecarboxylata* as a potential pathogen, even in non-immunosuppressed patients, and emphasized the need for further research on the pathogen’s resistance patterns and risk factors [9]. In contrast to Shaikhain et al.’s case [9], where *L. adecarboxylata* was identified through blood cultures in a non-immunosuppressed patient, our case required intraoperative cultures to isolate the pathogen from pleural fluid. The complexity of this case was further heightened by the presence of malignant mesothelioma, creating a unique scenario involving both a rare infectious pathogen and an underlying neoplastic condition. The association of *L. adecarboxylata* with empyema, particularly in immunocompromised patients, is scarcely documented in the literature, making this case especially significant for its rarity and the diagnostic challenges it posed.

Our case report describes a patient with immunosuppression due to corticosteroid use and mesothelioma, who developed a complicated parapneumonic effusion, progressing to empyema, caused by this rare opportunistic pathogen. This case contributes to the growing understanding of *L. adecarboxylata* as an emerging pathogen in respiratory infections and emphasizes the need for heightened clinical suspicion in similar cases. Furthermore, it underscores the challenge of managing infections caused by multidrug-resistant organisms, particularly in immunocompromised patients. By presenting this case, we aim to enhance diagnostic and therapeutic approaches for rare infections like *Leclercia adecarboxylata*-associated empyema, while maintaining patient privacy and confidentiality.

## 2. Case Presentation

A 57-year-old male patient with a history of type 2 diabetes mellitus and hypertension presented with cervical pain radiating to the chest and left upper limb, described as burning in nature. Initially, cervical radiculopathy was suspected, and a 30-day course of steroids (Dexamethasone 8 mg IV every 12 h) was prescribed. However, during further investigations by the spinal surgery team, pulmonary infiltrates and a left-sided unilocular pleural effusion were identified, which was not amenable to drainage. The patient was treated with piperacillin/tazobactam for six days. A chest CT scan also revealed a mass in the left apical region, leading to a pulmonary decortication performed by thoracic surgery, after which the patient was discharged.

He was readmitted to our institution 8 days later following pulmonary decortication and pleurodesis, presenting with pulmonary sepsis and systemic inflammatory response syndrome. Laboratory findings on readmission revealed significant abnormalities indicative of infection and systemic inflammation, as outlined in Table 1.

**Figure 1:**
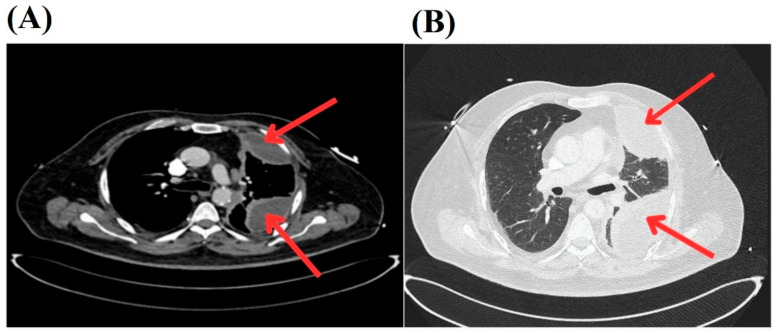
Chest CT images showing loculated empyema in the left hemithorax. (**A**) The red arrows indicate a loculated empyema with a small air component in the left hemithorax, resulting in near-total collapse of the lower lobe and partial collapse of the upper lobe. (**B**) The red arrows point to a loculated empyema in the left hemithorax with suspicion of a bronchopleural fistula, as evidenced by air pockets and fluid collection.

A subsequent chest CT scan revealed a “loculated empyema in the left hemithorax with a small air component, causing near-total collapse of the lower lobe and partial collapse of the upper lobe, with suspicion of an associated bronchopleural fistula” (see Figure 1).

As a result, the patient underwent pleurectomy and pleurodesis. Surgical findings revealed a multiloculated collection with fibrin clots, indicating superinfection, generalized lung entrapment, thickened parietal pleura, and cloudy fluid throughout the pleural cavity. Intraoperative cultures identified *Leclercia adecarboxylata* (see Table 2), underscoring the advanced stage of infection likely responsible for the patient’s worsening clinical condition. The combination of surgical intervention and targeted antibiotic therapy was crucial in managing the infection and preventing further complications.

Before starting empirical treatment, the intraoperative cultures obtained during the pleurectomy and pleurodesis allowed for the precise identification of *L. adecarboxylata* using the VITEK MS system by bioMérieux, with a 99.6% confidence level [10,11]. Although *L. adecarboxylata* is generally sensitive to a broad range of antibiotics—including tetracyclines, aminoglycosides, most beta-lactams (except penicillin G and oxacillin), quinolones, folate pathway inhibitors, chloramphenicol, nitrofurantoin, and azithromycin—it is naturally resistant to penicillin G, oxacillin, erythromycin, clarithromycin, ketolides, lincosamides, streptogramins, linezolid, glycopeptides, rifampicin, fusidic acid, and Fosfomycin [12,13].

Given the well-documented susceptibility profile of this pathogen and the rapid identification provided by the VITEK MS system, the medical team determined that the immediate initiation of targeted antibiotic therapy was appropriate without awaiting further susceptibility testing. Ampicillin/sulbactam was chosen based on its efficacy against *L. adecarboxylata* and its favorable pharmacokinetic properties for treating pleural infections. The patient’s clinical condition required prompt treatment, and his favorable response to ampicillin/sulbactam confirmed the appropriateness of this decision [12,14]. This direct identification likely eliminated the need for additional susceptibility testing, as the organism’s characteristics and resistance patterns are well-established in the literature [12,15].

This approach is supported by the existing literature indicating that most *L. adecarboxylata* infections can be effectively managed with empiric therapy guided by known sensitivity patterns. Therefore, additional antibiotic susceptibility testing was deemed unnecessary in this context. Antibiotic therapy was adjusted following recommendations from the infectious disease team, and the patient received intravenous ampicillin/sulbactam for 6 weeks. He showed satisfactory progress under home hospitalization, with the gradual resolution of symptoms and normalization of inflammatory markers [1,3,15,16].

Pathology and immunohistochemistry from an external institution revealed neoplastic cells in the pleura, positive for AE1/AE3 keratins, keratins 5/6, keratin 7, D2-40, GATA3, and calretinin, with a KI67 index of 8%, findings consistent with malignant mesothelioma of the epiploic variety. Following this, the patient was discharged under the care of the oncology department, where chemotherapy with Pemetrexed, Cisplatin, and Pembrolizumab was initiated to improve survival and control the progression of the malignant mesothelioma, achieving good symptomatic relief.

## 3. Discussion

This case report highlights the complexity and challenges associated with *L. adecarboxylata* infection, a rare pathogen that can manifest with various symptoms depending on the patient’s immune status. In immunocompetent individuals, the infection often appears as part of polymicrobial infections, leading to conditions such as skin and soft tissue infections, cholecystitis, peritonitis, or pneumonia [15]. In contrast, in immunocompromised patients, *L. adecarboxylata* infection is more likely to be monomicrobial, making diagnosis more challenging. The uniqueness of this case lies in the association of *L. adecarboxylata* with postoperative empyema in a patient with malignant mesothelioma which is a combination scarcely documented in the literature. This novel presentation emphasizes the need for heightened clinical awareness when diagnosing infections in immunocompromised patients, as unusual pathogens may be easily overlooked.

As previously noted, the rarity of *L. adecarboxylata*-associated postoperative empyema coexisting with malignant mesothelioma sets this case apart. Such a combination is noteworthy due to the limited reports of *L. adecarboxylata* causing empyema, particularly in high-risk, immunocompromised patients. The overlap of infectious and malignant processes required a comprehensive and nuanced approach to diagnosis and treatment, underscoring the complexity and diagnostic challenges posed by this case. Given the scarcity of similar presentations, our report contributes valuable information to the limited body of literature on *L. adecarboxylata*-associated infections in patients with cancer, thereby broadening the understanding of potential clinical manifestations and management strategies for this emerging pathogen.

The accurate identification of *L. adecarboxylata* in intraoperative cultures was pivotal to understanding the infection’s true nature. Utilizing the VITEK MS system by bioMérieux, which employs MALDI-TOF (Matrix-Assisted Laser Desorption/Ionization-Time of Flight) mass spectrometry, the pathogen was rapidly identified with 99.6% confidence. This method analyzes unique protein patterns in bacterial cell walls, offering a faster and more accurate identification compared to traditional culture-based methods that require extended incubation periods and may have limited sensitivity for less common pathogens [17,18]. Older systems, such as biochemical testing, often lack precision in distinguishing rare or emerging pathogens like *L. adecarboxylata*, potentially leading to misidentification as more common *Enterobacteriaceae* [19]. Therefore, the use of VITEK MS significantly contributed to rapid and accurate diagnosis, underscoring the value of modern, mass spectrometry-based systems in managing complex clinical cases involving unusual pathogens.

Genetic testing was not conducted in this case. This decision was based on the accurate identification provided by VITEK MS and the established sensitivity profile of *L. adecarboxylata*, which generally does not exhibit significant genetic variability affecting antibiotic response. While genetic testing can provide additional information on resistance mechanisms particularly in multidrug-resistant strains, such testing is typically reserved for cases where standard treatment fails or when there is a high suspicion of resistance genes impacting therapeutic choices [1,3,15,16].

Comparisons with previous reports further illustrate the variable antibiotic resistance profiles of *L. adecarboxylata*, which can depend on the strain and patient context. For instance, Botero-Garcia et al., 2018 [2], (see Table 3) reported a case involving a 69-year-old male with a soft tissue infection, where the isolated *L. adecarboxylata* strain was sensitive to a broad range of antibiotics including beta-lactams, aminoglycosides, and quinolones, but resistant to Fosfomycin [2]. Similarly, Mokkapati et al., 2014 [20], described a 31-year-old woman with *L. adecarboxylata* isolated from vaginal discharge, exhibiting multisensitivity but resistance to fosfomycin, highlighting the pathogen’s natural fosfomycin resistance. Furthermore, Matsuura et al., 2018, reported an 83-year-old patient with pneumonia caused by *L. adecarboxylata*, noting a similar resistance pattern [21]. These studies suggest that while *L. adecarboxylata* is often susceptible to a broad range of antibiotics, resistance to specific agents like fosfomycin is common and should be considered in treatment planning.

This case emphasizes the need for personalized antibiotic therapy based on pathogen characteristics and known resistance patterns, especially in immunocompromised patients [19]. Despite the successful outcome, this case report has certain limitations. The absence of genetic testing for *L. adecarboxylata* restricts our ability to assess potential underlying resistance mechanisms that may not be apparent in standard susceptibility profiles. While the rapid identification and known sensitivity patterns supported an effective treatment approach, the growing incidence of multidrug-resistant strains of *L. adecarboxylata* suggests that genetic testing may become increasingly valuable for comprehensive resistance analysis. Additionally, reliance on literature-based susceptibility patterns, although effective here, may not always capture the variability in resistance seen across different settings and patient populations [12,13].

Therefore, future studies should explore the role of genetic testing in cases involving *L. adecarboxylata*, particularly to identify resistance genes that could impact treatment decisions. Establishing standardized guidelines for managing infections with emerging and rare pathogens, especially in immunocompromised patients, would also be beneficial. This could include recommendations for genetic or molecular analysis in complex cases where resistance profiles are unclear, thereby improving tailored therapeutic strategies and patient outcomes.

## 4. Conclusions

This case report highlights the importance of the early detection and management of *L. adecarboxylata* infections in immunosuppressed patients, who may present with atypical infections, as demonstrated by this case of empyema associated with a parapneumonic effusion. Since this bacterium is rarely diagnosed in clinical practice, it is essential to consider its presence in patients with complex risk factors, including chronic conditions and immunosuppression. Delayed diagnosis can significantly increase the risk of antimicrobial resistance. Therefore, conducting antibiotic sensitivity tests is crucial to tailor treatment individually, taking into account the patient’s underlying conditions and immune status.

These findings emphasize the importance of a multidisciplinary approach to managing opportunistic infections in vulnerable patients. Future studies are needed to establish clearer therapeutic guidelines and improve early identification of this bacterium in high-risk clinical settings. This approach reinforces the necessity for personalized clinical management and could serve as a foundation for future research into underdiagnosed infections and the development of more effective treatment strategies.

## Figures and Tables

**Table 1:** Blood test results.

Test	Results	Reference Value
Sodium	124.8 mEq/L	135–145 mEq/L
Magnesium	1.16 mg/dL	1.7–2.2 mg/dL
Chloride	87.4 mEq/L	98–106 mEq/L
Phosphorus	1.82 mEq/L	2.5–4.5 mEq/L
Leukocytes	18,390/mm^3^	4000–11,000/mm^3^
Lymphocytes	1970/mm^3^	1000–4800/mm^3^
Neutrophils	15,640/mm^3^	1500–8000/mm^3^
Hemoglobin	13.3 g/dL	13.5–17.5 g/dL (Males)
BUN *	16.1 mg/dL	7–20 mg/dL
pH	7.46	7.35–7.45
PO_2_ *	87.0 mmHg	75–100 mmHg
Lactic Acid	2.48 mmol/L	0.5–2.2 mmol/L
CRP *	269.7 mg/L	<10 mg/L

* Description: BUN: blood urea nitrogen; CRP: C-reactive protein; PO_2_: partial pressure of oxygen.

**Table 2:** Culture results.

Test	Results
Blood cultures	Negative after 5 days
Urine culture	Negative
Intraoperative culture	*L. adecarboxylata*

**Table 3:** Comparative study of clinical presentation, procedures, risk factors, and treatment in cases of *L. adecarboxylata*.

Author	Age and Gender	Medical History	Clinical Manifestation	Procedure Performed	Treatment
C.A. Botero-García et al., 2018 [2]	69-year-old male	Metabolic syndrome	Soft tissue lesion, mild purulent IDSA	Culture and isolation of germ from lesion	Ampicillin-sulbactam 6 g every 6 h for 10 days
Dr. Mokkapati et al., 2014 [20]	50-year-old male	No significant medical history	Soft tissue infection, mild IDSA	Drainage and culture of lesion	Doxycycline 100 mg every 12 h for 14 days
Dr. Mokkapati et al., 2014 [20]	31-year-old female	No significant medical history	Malodorous vaginal discharge	Vaginal discharge culture	Doxycycline 100 mg every 12 h
Dr. H. Matsuura et al., 2018 [21]	83-year-old male	Comorbidities, low-grade prostate cancer	Respiratory symptoms progressing to SIRS, healthcare-associated pneumonia, severe soft tissue infection	Blood cultures, tissue cultures	Not reported

## Data Availability

Data are contained within the article.
